# Supplementary material for: Sexual dysfunction in patients with diabetes: association between remnant cholesterol and erectile dysfunction
Source: Lipids Health Dis. 2024 Feb 22;23:55. doi: 10.1186/s12944-024-02046-8 (PMC10882869; doi:10.1186/s12944-024-02046-8)
Supplement: Supplementary file 1 — Supplementary Material 1 [file 12944_2024_2046_MOESM1_ESM.pdf]

PAPER NAME

0212232135981.docx

WORD COUNT	CHARACTER COUNT
2921 Words	17348 Characters
PAGE COUNT	FILE SIZE
14 Pages	123.9KB
SUBMISSION DATE	REPORT DATE
Feb 12, 2024 11:23 PM GMT+8	Feb 12, 2024 11:23 PM GMT+8

9% Overall Similarity

The combined total of all matches, including overlapping sources, for each database.

- 6% Internet database
- 7% Publications database
- Crossref database
- Crossref Posted Content database

Excluded from Similarity Report

- Bibliographic material

Sexual dysfunction in patients with diabetes: association between remnant cholesterol and erectile dysfunction

Abstract

Background: Erectile dysfunction (ED) is closely associated with dyslipidemia; however, it is yet unknown how ED and remnant cholesterol (RC) are related. As such, this research sought to explore the correlation between RC and ED among individuals with diagnosed with diabetes.

Methods: This cross-sectional study used information from 215 males from National Health and Nutrition Examination Survey (NHANES) from 2001 to 2004. RC was calculated as follows: the values of high-density lipoprotein cholesterol (HDL-c) and low-density lipoprotein cholesterol (LDL-c) were subtracted from the total cholesterol (TC) value, while ED diagnoses were based on self-reports. Weighted logistic regression analyses using both univariate and multivariate approaches were conducted to assess the correlation between RC and ED.

Results: After comprehensive adjustment, multivariable logistic regression models revealed a strong correlation between RC and ED in subjects with diabetes (with an odds ratio (OR) of 7.49 and a 95% confidence interval (CI) of 1.98–28.37; *P*=0.004). On categorizing RC into 3 grades (T1-T3), the OR corresponding to higher RC grade increased. Despite the results not reaching statistical significance upon categorization, a consistent and statistically significant trend (*P* for trend < 0.05) was observed.

Conclusion: This study indicated a correlation between increased RC levels and a

higher prevalence of ED in diabetic males. RC may serve as a promising predictor of ED in individuals with diabetes. However, additional studies are required to confirm these findings.

Keywords: Remnant cholesterol, Erectile dysfunction, Diabetes, NHANES.

1. Background

Erectile dysfunction (ED) has been observed to have an increasing incidence with advancing age[1]. ED is currently acknowledged as a serious health concern among the growing population, leading to diminished quality of life for individuals affected, as well as for their partners and families[2]. The National Institutes of Health's commonly used definition of ED is the inability to develop or maintain an erection firm enough for sexual intercourse[3]. There are many risk factors, including systemic disease(s), and diabetes may increase the incidence of ED[4, 5], with some clinical and epidemiological research supporting a link between ED and metabolic syndrome (MS)[6, 7]. MS is a multifaceted disease that imposes considerable socioeconomic burdens, and mainly includes hypertension, dyslipidemia, and impaired blood glucose regulation. In addition, it is increasingly acknowledged that insulin resistance (IR) and abdominal obesity are the main signs of MS[8]. Thus, it is crucial and beneficial that further research be done on the risk factors related to ED.

Extensive research has revealed a correlation between hyperlipidemia/dyslipidemia and ED[9]. Furthermore, several epidemiological investigations have indicated that serum markers of dyslipidemia, such as the ratio of

high-density lipoprotein cholesterol (HDL-c) to total cholesterol (TC) and levels of HDL-c and low-density lipoprotein cholesterol (LDL-c) serve as predictors of ED[10-12]. Therefore, the influence of cholesterol levels, other than HDL-c and LDL-c, on the occurrence of ED merits further investigation. Most of the remaining cholesterol is primarily composed of intermediate-density lipoproteins, very low-density lipoproteins, and remnants of chylomicrons, which are also known as remnant cholesterol (RC)[13]. It circulates within the plasma and accumulates in the subendothelial region[14, 15], leading to endothelial dysfunction, inflammation, and eventually the onset of atherosclerosis[15, 16].

Diabetes poses a significant and escalating global health burden, with an estimated 592 million individuals worldwide projected to be affected by the condition by 2035[17, 18]. Complications of diabetes mellitus (DM) present a substantial healthcare challenge, encompassing macroangiopathy, microangiopathy, and sexual dysfunction in both sexes. In males with DM, the incidence of ED is roughly 3.5 times higher than in those without the disease[19]. Patients with DM frequently have hyperlipidemia, which leads to the greater risk of vascular disease in this population[20]. Individuals with DM exhibit elevated RC levels compared to those without DM. It has been found that elevated RC levels and low-grade inflammation have been linked to a higher likelihood of atherosclerotic cardiovascular disease in participants diagnosed with DM[21]. According to a prior study, ED could serve as a standalone predictor for cardiovascular disease (CVD)

and its associated outcomes[22]. Therefore, there may be a close correlation between RC and the development of ED among individuals with DM.

To the best of current knowledge, there is limited research investigating the relationship between RC and ED in males with DM. For the first time, a novel correlation between RC and ED may be identified, particularly within the diabetic population, which may help provide different approaches to ED intervention or prevention. Therefore, to explore the question, this cross-sectional investigation used extensive information from the National Health and Nutrition Examination Survey (NHANES) (2001-2004).

2. Materials and method

2.1 Study population

The Centers for Disease Control and Prevention administers the NHANES, a survey that attempts to assess American health and nutritional status. The NHANES has a multi-stage, complicated probability sampling approach. Every year, the program investigates a nationally representative sample of five thousand individuals drawn from 15 different sites within a sampling frame of all counties in the United States[23]. The survey aimed to analyze a range of 200 to 400 individuals in each of the sampling domains, which varied from 60 to 80, encompassing various demographic factors including age, sex, race, and ethnicity, over a comprehensive four-year survey period[24]. This survey is unique because it integrates interviews and physical assessments. The interviews address topics including demographics, socioeconomic factors, dietary habits, and health-related information. The survey

includes a range of dental, medical, and physiological assessments performed by skilled medical personnel along with laboratory analyses. Authorization for use of the survey was received from the National Center for Health Statistics Research Ethics Review Board (NCHS REB), which guaranteed that each participant provided informed consent. The website provided access to extensive statistical data(https://www.cdc.gov/nchs/nhanes/).

Due to the restricted availability of questionnaire information addressing ED in other years, in this study, NHANES data from two cycles (2001–2002 and 2003–2004) were used. Of 31,473 samples, only 7261 remained for analysis due to the absence of cholesterol data for 24,482 samples. Further exclusion of 5455 samples with missing ED data resulted in the inclusion of 1806 samples. Individuals without DM or with incomplete information were excluded (n=1591). Ultimately, a cohort comprising 215 individuals was included in this study (**Figure 1**).

2.2 Assessment of ED

The subsequent inquiry, taken from the Massachusetts Male Aging Study, was used to evaluate erectile function, “Many men have difficulties during sexual activity. What would you say about your capacity to achieve and maintain an erection strong enough for fulfilling sexual activity?”. “Usually able”, “always or almost always able”, “sometimes able”, and “never able” were the options for the responses. “The inability to sustain an erection sufficient to satisfy sexual relations was defined as ED[25, 26].

2.3 Assessment of RC

In the mobile examination center (MEC), the samples of blood are prepared, kept, and then sent to the Lipoprotein Analytical Laboratory at Johns Hopkins University for analysis. The RC value was derived by subtracting the combined levels of LDL-c and HDL-c from the TC level, which was determined based on the patient's standard fasting lipid profile[27, 28].

2.4 Assessment of DM

Subjects in this study were diagnosed with DM. Subjects were considered to have DM if physicians had informed them of their condition, current usage of glucose-lowering medication, or glycated hemoglobin (HbA1c) levels $\geq 6.5\%$.

2.5 Covariates

Based on previous research, potential factors considered in the study encompassed age, race/ethnicity, body mass index (BMI), level of education, smoking and alcohol consumption status, hypertension, and CVD. Five separate groups were created based on self-reported racial/ethnic identity: Mexican Americans; Other Hispanic; Non-Hispanic White; Non-Hispanic Black; and Other Race-Including Multi-Racial. Educational levels were categorized into five groups: < 9th grade; 9–11th grade, high-school graduate/GED or equivalent; college or AA degree; and college graduate or above. Individuals who acknowledged consuming a minimum of 12 alcoholic beverages throughout their lifetime were categorized as having a drinking habit. Those who had ever smoked $\geqslant 100$ cigarettes were classified as smokers for the purposes of the survey. Individuals who were surveyed and found to have hypertension had been previously diagnosed with the condition.

Additionally, participants identified with myocardial infarction, angina, coronary artery disease, or heart failure were categorized as having CVD.

2.6 Statistical analysis

All statistical analyses were conducted using EmpowerStats (X&Y Solutions, Inc. <http://www.empowerstats.com>) and R (The R Foundation <http://www.R-project.org>). Due to the intricate sampling design used in the NHANES, strata, primary sampling units, and sampling weights were taken into account during the data analyzing process to ensure that the calculated statistics accurately represented the entire US population, following the National Center for Health Statistics' guidelines[29, 30].

In the baseline features(Table 1), continuous variables are represented as weighted mean and standard deviation (SD), whereas categorical data are expressed as weighted proportion. Survey-weighted linear regression was employed to examine differences between individuals with and without ED for continuous variables, whereas survey-weighted chi-squared tests were employed to analyze categorical variables. Logistic regression models, both univariate and multivariate, were used to investigated the correlation between the prevalence of ED and RC level in patients with DM. Three alternative models were used in this study: Model 1 (unadjusted); Model 2, which considered age, BMI, and race/ethnicity; and Model 3, which included adjustments for Model 2 + education level, smoking status, alcohol intake, hypertension, and CVD.

3. Results

3.1 Basic characteristics

A sample of 215 subjects 20 to 85 years of age, was included in the study. Of these, 61 reported experiencing ED, while the remaining 154 did not. Most subjects with ED were > 40 years of age. The largest percentage of subjects with ED had academic degrees, comprising college graduates or above (27.9%). The incidence of ED was considerably higher among smokers (75.4%) compared with nonsmokers (66.2%). Among those who reported ED, 44.3% had a BMI > 30 kg/m^2. As weight increased, from normal weight to overweight to obese, the prevalence of ED increased. Of those who reported ED, 63.9% reported a history of hypertension, and 47.5% had CVD.

3.2 The association between RC and ED

Statistical analysis of the stratified samples revealed a notable correlation between RC and ED among subjects with DM, suggesting that RC may put those with DM at risk for developing ED. ED is more common among individuals with elevated RC levels. The relationship between RC and ED in subjects diagnosed with DM was investigated using multivariate logistic regression analysis that was weight-adjusted. Using the unadjusted model, partially adjusted variables, and a fully adjusted model, a notable association was detected between RC and ED. Detailed information is summarized in Table 2. In the unadjusted model (i.e., Model 1) (odds ratio (OR) 2.86 [95% confidence interval (CI) 1.37–5.95]; *P*=0.009), partially adjusted variables (Model 2) (OR 4.14 [95% CI 1.76–9.74]; *P*=0.004), and completely adjusted model (Model 3) (OR 7.49 [95% CI 1.98–28.37]; *P*=0.007), the

association between ED and RC remained strongly positive despite adjustment for more variables. In addition, on categorizing RC into 3 grades (T1-T3), an increase in OR values corresponding to higher RC grade was observed. Although the outcome did not achieve statistical significance following categorization, a consistent and statistically significant trend was observed (*P* for trend < 0.05).

4. Discussion

The purpose of this study was to evaluate the connection between RC and ED in subjects diagnosed with DM. Data from two NHANES cycles (2001-2004) were retrieved and analyzed. Findings revealed a positive relationship between RC and the prevalence of ED in subjects with DM, suggesting that RC may, in part, represent a risk factor for ED.

Hyperlipidemia/dyslipidemia, an important component of MS, significantly contributes to vascular risk and affects erectile function. This effect may be attributed to the development of atherosclerosis and damage to the endothelium[9]. According to some studies, each MS component is crucial for diagnosing ED and, treating each component separately is vital[31]. Research has also found that individuals with obstructive sleep apnea syndrome (OSAS) experience an increased likelihood of developing ED[32]. Due to intermittent hypoxia caused by OSAS, some researchers have speculated that this disturbance in circadian rhythm may result in reduced nitric oxide (NO) production, altered sex hormone levels, and neurological dysfunction, all of which could impair erectile function[33, 34].

Furthermore, several investigations have linked elevated RC levels to CVD, and

RC is independent of traditional cardiovascular risk factors[35]. Moreover, some evidence suggests that ED is also a distinct indicator of CVD; alternatively, ED causes CVD by initiating related CVD events[36], and ED and CVD represent two manifestations of an underlying shared physiological process[22]. Therefore, many risk factors associated with CVD may increase the possibility of developing ED. Piero Montorsi et al. found that all vascular beds are equally affected by atherosclerosis; however, compared to bigger arteries, smaller arteries are more likely to become blocked[37, 38]. Compared to the coronary arteries, the penile arteries are smaller in diameter. Consequently, similar to coronary arteries, penile arteries are more prone to have blood flow obstruction at the same degree of endothelial dysfunction and atherosclerosis[37]. Glass CK et al. reported that elevated plasma cholesterol levels may independently drive the development of atherosclerosis[39]. It is considered that RC may be a major contributor in the development of atherosclerosis through its pro-inflammatory effects, which include the induction of low-grade inflammation and the activation of endothelial cells in a manner that promotes inflammation[40]. One hypothesis proposed a potential connection between heightened RC levels and increased triglyceride content of LDL-c particles, particularly the small dense LDL-c particles that tend to promote atherosclerosis and have a prolonged residence time in the bloodstream[41]. Surprisingly, a strong correlation has been found between increased RC levels and a five-fold rise in the likelihood of peripheral artery disease in the general population[42]. Hence, there is a potentially close relationship between RC and ED.

The key to future treatments may rely on switching to a different strategy that considers the unique constituents of non-HDL-c, which could lead to more targeted and effective interventions; clinical trials are already assessing reducing treatments that target RC[43]. Therefore, based on results of this study and extensive existing researches, it is possible that the involvement of RC could potentially impact the subsequent development and advancement of ED, and this study may contribute to providing new directions for the management and prevention of ED. However, further investigation is crucial to determine the mechanism(s) and significance.

Moreover, DM is associated with higher RC levels, which are likely attributable to dietary and lifestyle variables, as well as IR and high blood glucose levels, which lead to greater retention of triglyceride-rich lipoproteins in the plasma, impairing the activity of lipoprotein lipases[44, 45]. Reactive oxygen species (ROS) may accumulate up in pancreatic β-cells due to stress in the endoplasmic reticulum and malfunction in the mitochondria caused by an excess of cholesterol. Consequently, this process may lead to structural modifications in particles containing insulin[46]. The abundance, size, and cholesterol content of RC particles surpasses those of LDL-c particles. Therefore, it is possible to speculate that RC poses a greater risk to pancreatic β-cells[47]. Additionally, RC may exacerbate IR and induce a systemic proinflammatory state, potentially leading to aberrant glucose metabolism[48,49]. Research has revealed a strong correlation between IR and Peyronie’s disease[50], which is considered to be a cause of ED. Meanwhile, ED may be exacerbated by IR states, which are characterized by reduced vascular NO production and diminished

insulin-induced vasodilation[51].The impact of DM on ED encompasses various elements such as hormonal, vascular, and neurological factors[52]. Traditionally, the fundamental cause has been attributed to microvascular impairment and inadequate delivery of oxygen and blood supply to nerves due to glycosylation[53]. Furthermore, endothelial dysfunction caused by ROS and hyperglycemia can reduce vascular NO levels and impair vasodilation[54, 55], thereby significantly contributing to the increased incidence of ED in patients with DM. Therefore, it is essential to prevent and treat ED by effectively managing DM and RC levels. Consequently, in the context of diabetic ED, future research could focus on elucidating the mechanistic link between elevated RC levels, inadequate glycemic management, persistent low-grade inflammation, and IR.

5. Strengths and limitations

This study boasts several advantages. Notably, A sizable sample taken from the NHANES was used in this thorough analysis of diabetes patients in the United States. Second, little research has investigated the relationship between RC and ED, especially among individuals with DM. Third, the pathogenesis of ED is complex, and the identification of new risk factors is advantageous for its early detection and management; consequently, prospects for clinical application(s) are promising. However, the study also had some limitations, the first of which was its cross-sectional design, which pose limitations when assessing the association between fluctuations in RC levels and the onset of ED. Therefore, a direct causal relationship between RC and ED could not be inferred from the findings. As such, further high-

quality prospective studies are required to obtain more conclusive results. Additionally, ED assessment is based on patient self-reports, which, while convenient and noninvasive, introduce the potential for recall bias. Standardized assessment tools or medical records are options to mitigate the effects of recall bias; however, the cost involved in such research is high. Therefore, additional studies to establish a causal link between RC and the prevalence of ED in diabetic individuals are warranted.

6. Conclusion

This study found that among males with DM, there was a positive connection between elevated RC levels and a higher prevalence of ED. RC could potentially act as a valuable predictor for ED in individuals with DM, thus enabling clinicians to implement more targeted screening and intervention strategies for ED. However, the causal connection between RC and ED necessitates additional research.

1

9% Overall Similarity

Top sources found in the following databases:

- 6% Internet database
- 7% Publications database
- Crossref database
- Crossref Posted Content database

TOP SOURCES

The sources with the highest number of matches within the submission. Overlapping sources will not be displayed.

1	mdpi.com<br>Internet	<1%
2	assets.researchsquare.com<br>Internet	<1%
3	worldwidescience.org<br>Internet	<1%
4	Liuyang Li, Julie M. Schallhorn, Jiaonan Ma, Lin Zhang, Rui Dou, Yan W...<br>Crossref	<1%
5	science.gov<br>Internet	<1%
6	D Justo. "Increased erythrocyte aggregation in men with coronary arter...<br>Crossref	<1%
7	medrxiv.org<br>Internet	<1%
8	David F Mobley, Mohit Khera, Neil Baum. "Recent advances in the treat...<br>Crossref	<1%
9	kkbrum.r-universe.dev<br>Internet	<1%

10	link.springer.com<br>Internet		<1%
11	Lan Chen, Lijun Chen, Han Zheng, Sunying Wu, Saibin Wang. "The asso...<br>Crossref		<1%
12	stacks.cdc.gov<br>Internet		<1%
13	tandfonline.com<br>Internet		<1%
14	articlealley.com<br>Internet		<1%
15	DeFilippis, Andrew Paul, Michael J. Blaha, Seth S. Martin, Robert M. Re...<br>Crossref		<1%
16	Yukari Okawa, Toshiharu Mitsuhashi. "Association between blood pres...<br>Crossref posted content		<1%
17	Storti, K.L.. "Physical activity and decreased risk of clinical gallstone di...<br>Crossref		<1%
18	cdc.gov<br>Internet		<1%
19	dovepress.com<br>Internet		<1%
20	pdr.net<br>Internet		<1%
21	Cheng, Lim Yin. "Health Effects of Night-Shift Work Among Manufactur...<br>Publication		<1%

22	Matthew Untalan, Tara Ivic-Pavlicic, Emanuela Taioli. "Urinary Glyphos...	Crossref	<1%
23	Weilong Ding, Jing Wang, JunBao Yang, XiangYu Wang. "High levels of ...	Crossref posted content	<1%
