# Supplementary material for: Sexual dysfunction in patients with diabetes: association between remnant cholesterol and erectile dysfunction
Source: Lipids Health Dis. 2024 Feb 22;23:55. doi: 10.1186/s12944-024-02046-8 (PMC10882869; doi:10.1186/s12944-024-02046-8)
Supplement: Supplementary file 2 — Supplementary Material 2 [file 12944_2024_2046_MOESM2_ESM.pdf]

Editing Certificate

This document certifies that the manuscript listed below has been edited to ensure language and grammar accuracy and is error free in these aspects. The edit was performed by professional editors at Editage, a brand of Cactus Communications. The author's core research ideas were not altered in any way during the editing process. The quality of the edit has been guaranteed, with the assumption that our suggested changes have been accepted and the text has not been further altered without the knowledge of our editors.

MANUSCRIPT TITLE

Sexual dysfunction in patients with diabetes: association between remnant cholesterol and erectile dysfunction

AUTHORS

Ke Huang

ISSUED ON

February 07, 2024

JOB CODE

TJBTD_1

THE EDITAGE Promise QUALITY GUARANTEE

Prabh Grewal
Senior Vice President - Editage

editage helping you get published

Since 2002, Editage has helped over 430,000 authors publish around 1.2 million research papers in scholarly journals across over 1000 disciplines through editorial, translation, transcription, and publication support services. Editage is a brand of Cactus Communications (cactusglobal.com), a science communication and technology company.

CACTUS.

GLOBAL :
+1(833) 979-0061 | request@editage.com

CHINA :
400-120-3020或:021-6020-9400 |
fabiao@editage.cn
